# Curcumin promotes osteogenic differentiation of periodontal ligament stem cells through the PI3K/AKT/Nrf2 signaling pathway

**DOI:** 10.22038/IJBMS.2020.44070.10351

**Published:** 2020-07

**Authors:** Yixuan Xiong, Bin Zhao, Wenjing Zhang, Linglu Jia, Yunpeng Zhang, Xin Xu

**Affiliations:** 1 School of Stomatology, Shandong University, Jinan, China; 2 Shandong Provincial Key Laboratory of Oral Tissue Regeneration, Jinan, China; 3 Department of Oral Implantology, the Affiliated Stomatology Hospital of Kunming Medical University, Kunming, China

**Keywords:** AKT, Curcumin, Nrf2, Osteogenic differentiation, Periodontal ligament, Stem cells

## Abstract

**Objective(s):**

The aim of this study was to investigate the effect of curcumin on the osteogenic differentiation of human periodontal ligament stem cells (hPDLSCs) and its underlying potential mechanism.

**Materials and Methods:**

The tissue explant adherence method was used to isolate hPDLSCs. Flow cytometry, Alizarin Red staining and Oil Red O staining were applied to confirm the stemness of the stem cells. CCK8 assays were used to evaluate the effect of curcumin at different concentrations on cytotoxicity, and alkaline phosphate (ALP) activity assays, ALP staining and Alizarin Red staining were used to measure the osteogenic differentiation ability. In addition, hPDLSCs were treated with LY294002 (a phosphatidylinositol-3-kinase [PI3K] inhibitor) and erythroid transcription factor NF-E2 siRNA (siNrf2), respectively in the presence of curcumin. Western blotting was applied to evaluate the protein kinase B (AKT) phosphorylation levels and the Nrf2 levels. Besides, western blotting, RT-qPCR, ALP activity assays, ALP staining and Alizarin Red staining were used to detect the potential effects of curcumin on osteogenic differentiation.

**Results:**

Curcumin at an appropriate concentration had no cytotoxicity and could promote osteogenic differentiation of the hPDLSCs. The results of western blotting and RT-qPCR revealed that the protein and mRNA levels of ALP, COL1 and RUNX2 were increased by curcumin, while the PI3K/AKT/Nrf2 signaling pathway was activated. In addition, LY294002 was added to inhibit the PI3K/AKT signaling pathway, or siNrf2 was used to block the Nrf2 pathway; then, the stimulatory effects of curcumin on osteogenic differentiation were reversed.

**Conclusion:**

Curcumin could promote the osteogenesis of hPDLSCs, and the effect is related to the PI3K/AKT/Nrf2 signaling pathway.

## Introduction

Periodontal disease is a common and frequent oral inflammatory disease. Overabsorption of alveolar bone caused by periodontitis has negative effects on oral implantation. How to effectively restore periodontal tissue has been the focus of current research (1). Human periodontal ligament stem cells (hPDLSCs) have shown good potential in the process of periodontal tissue regeneration, which makes them a hot topic in the field of periodontal regeneration medicine (2). How to utilize hPDLSCs effectively and increase their use in the field is the focus of our research.

Researchers have shown that therapeutic agents influence cellular responses to achieve various biological functions, such as cell adhesion, proliferation and differentiation (3). Natural plant products have been used for various purposes throughout human history (4). Curcumin, a polyphenolic phytochemical derived from Indian dietary fibre spices, has been widely used for centuries in the treatment of various diseases due to its wide-ranging pharmacological activities (5, 6). Notably, curcumin has been found to be nontoxic and safe in research and clinical applications (7, 8). Recent studies have indicated that curcumin plays a pivotal role in the regulation of cell differentiation. For neural progenitor cells, curcumin could strongly affect the proliferation and differentiation, as well as the generation, synaptogenesis and migration of effective nerves (9, 10). Besides, curcumin could induce embryonic stem cell (ESC) differentiation by significantly promoting the expression of cardiac-specific transcription factors and cardiac-specific proteins (11). Moreover, the effect of curcumin on osteogenic differentiation has received increasing attention. Previous studies showed that curcumin could be used to reduce bone loss, which could be achieved by inhibiting the proliferation and differentiation of osteoclasts while promoting their apoptosis (12-14). In addition, curcumin could promote the osteogenic differentiation of mesenchymal stem cells (MSCs) and regulate bone formation (15). Based on previous studies, we speculate that curcumin can promote the osteogenic differentiation of hPDLSCs.

The phosphatidylinositol-3-kinase (PI3K)/protein kinase B (AKT) axis was first found in cancer cells, in which various functions are related to this signaling pathway (16). The signaling pathway is activated by various extracellular stimuli and regulates a wide range of cellular processes, including cell movement, survival, and proliferation and the cell cycle (17). The PI3K/AKT signaling pathway is essential for cellular differentiation. AKT is important in osteogenic differentiation and homeostasis (18). *In vivo* evidence has shown that severe growth deficiency and impaired bone development were found in AKT1/AKT2 double knockout (DKO) mice (19, 20). In addition, in some *in vitro* studies, the expression of osteogenic genes was significantly upregulated by activating the PI3K/AKT signaling pathway, and blocking this pathway produced the opposite effects (21). Some researchers proved that curcumin was an effective activator of erythroid transcription factor NF-E2 (Nrf2) (22, 23). In recent years, the role of Nrf2 in stem cell-specific differentiation and maintenance has also been emphasized, and it plays a crucial role in bone homeostasis (24). Further studies showed that activation of AKT signaling could induce the activation of Nrf2 (25). Based on the above studies, our research focused on whether curcumin can enhance the osteogenic differentiation of hPDLSCs and whether the effect was related to PI3K/AKT/Nrf2.

## Materials and Methods

***Cell isolation and culture***

Healthy bicuspid teeth extracted from 15- to 23-year-old patients due to orthodontics were obtained with the consent of the patients and their families. All clinical trials were approved by the Ethics Committee of Stomatology Medical College of Shandong University. Fresh extracted teeth were washed with phosphate-buffered saline (PBS) containing 5% penicillin/streptomycin (Beyotime, China), and the periodontal ligament at the middle one-third of the root was scraped and cut into fragments less than 1 mm in diameter and then attached to the bottom of the culture bottle. The culture medium contained minimum essential medium-α (α-MEM) (Gibco, USA), 20% foetal bovine serum (FBS) (Gibco, USA) and 1% penicillin/streptomycin. After 24 hr, the bottle was turned over. Cells were cultured in incubators at 5% carbon dioxide and 95% humidity. The medium was replaced every 3 days after the short spindle cells climbed out of the tissue mass. When the cells reached 80% confluence, they were passaged, and the P3-P5 generation was used in further experiments.

***Clonogenic experiment***

hPDLSCs were inoculated in a large dish at a density of 1000 cells in α-MEM containing 10% FBS, and the medium was replaced every 3 days. Fourteen days later, the hPDLSCs were washed with PBS, fixed with polyformaldehyde for 20 min and stained with crystal violet (Solarbio, China). Cell clones were detected under a microscope, and more than 50 cells were defined as a clone.

***Flow cytometry***

Flow cytometry was used to detect the surface markers of the hPDLSCs. The undifferentiated hPDLSCs were digested with trypsin and counted. The same number of cells were resuspended in FACS buffer (PBS containing 2% FBS). The following antibodies were used: CD90 FITC, CD44 PE, CD105, PerCP-Cy, CD73 APC and a PE-negative cocktail (CD34 PE, CD11b PE, CD19 PE, CD45 PE and HLA-DR PE). Then, flow cytometry (BD Biosciences) was used to detect the signals of labelled cells, and Flow Jo software (Beckman Coulter, Fullerton, CA, USA) was used to analyse the data.

***Cell viability***

hPDLSCs were inoculated into 96-muliwell plates at a density of 2×10^3^ cells in α-MEM containing 10% FBS and allowed to adhere overnight. Medium with different concentrations of curcumin was added to the 96-well plate. After 24, 48 and 72 hr of treatment, α-MEM containing 10% Cell Counting Kit-8 (CCK8) (Dojindo, Kumamoto, Japan) was used to replace the medium, and the absorbance at 450 nm was measured after incubation for 2 hr.

***Multidirectional differentiation***

hPDLSCs were cultured at a density of 2×10^5^ cells in 6-multiwell plates overnight, and α-MEM with 10% FBS was replaced by osteogenic induction medium (10% FBS, 50 mg/l ascorbic acid (Sigma–Aldrich, USA), 10 mM β-glycerophosphate (Sigma–Aldrich, USA), and 10^−8^ M dexamethasone (Sigma–Aldrich, USA)). After 7 days of treatment, an alkaline phosphate (ALP) kit (Beyotime, China) was used to detect the early osteogenic potential. In addition, 21 days later, Alizarin Red staining was used to detect the formation of mineralized nodules.

hPDLSCs were seeded at a density of 2×10^5^ cells in 6-multiwell plates overnight, and α-MEM with 10% FBS was replaced by adipogenic induction medium (10% FBS, 0.5 mM isobutyl-methylxanthine (Sigma–Aldrich), 2 μM dexamethasone (Sigma–Aldrich, USA), 0.2 mM indomethacin (Sigma–Aldrich), and 10 mg/l insulin (Sigma–Aldrich)). After 28 days, lipid droplets were detected by Oil Red O staining.

***ALP activity***

hPDLSCs were cultured in 6-multiwell plates at a density of 2×10^5^ cells in α-MEM with 10% FBS. After reaching 80% confluence, the cells were exposed to osteogenic induction medium. Seven days later, the cells were collected for detection of the total intracellular protein concentration and ALP activity (Nanjing Jiancheng, Nanjing, China). The final absorbance of the total protein was detected at 562 nm. The ALP activity was detected at 520 nm. The ALP levels were standardized to the total protein contents.

***siRNA and vector transfection***

hPDLSCs were cultured in 6-well plates at a density of 2×10^5^ cells in α-MEM with 10% FBS and transfected with Nrf2 siRNA or control siRNA using a transfection reagent (Micropoly, China) according to the manufacturer’s protocols. After 24 hr of incubation, the transfected cells were used for the subsequent experiments. Western blotting was used to detect the transfection efficiency. The siRNA sequences used in the experiment were as follows: siRNA targeting Nrf2 (siNrf2, forward 5’-GCCCAUUGAUGUUUCUGAUTT-3’ and reverse 5’-AUCAGAAACAUCAAUGGGCTT-3’) and scramble nonsense siRNA (scramble control, forward 5’-UUCUCCGAACGUGUCACGUTT-3’ and reverse 5’-ACUUGACACGUUCGGAGAATT-3’).

***Western blotting***

Total cytoplasmic and nuclear proteins were extracted using lysis buffer (Solarbio, China) containing 1% protease and 1% phosphatase inhibitor (Boster, Wuhan, China), and a bicinChoninic acid (BCA) protein assay kit (Solarbio, China) was used to determine the protein concentration. Equal amounts of protein were then separated by SDS-PAGE and transferred to PVDF membranes. The membrane was incubated overnight at 4°C with primary antibodies against ALP (1:20000, ab108337; Abcam), COL1A1 (1:1000, #84336; Cell Signaling Technology), RUNX2 (1:1000, Cell Signaling Technology), p-AKT (1:1,000, #4060; Cell Signaling Technology), AKT (1:1000, #4691; Cell Signaling Technology), Nrf2 (1:1000, ab62352; Abcam), GAPDH (1:20000, HRP-60004; Proteintech), and Histone H3 (1:1000, Ab1791; Abcam), and then, secondary antibodies were incubated with the membrane for 1 hr at room temperature. Chemiluminescence HRP (Millipore) was used to detect the protein bands. ImageJ 1.47V was used to analyse the protein levels.

***Real-time quantitative PCR***

Total RNA was extracted by Trizol (TaKaRa, Tokyo, Japan) and then reverse transcribed to cDNA using a Super Script TM II reverse transcriptase kit (TaKaRa, Tokyo, Japan) following the instructions. Next, real-time quantitative PCR was performed using SYBR^®^ Primix Ex Taq™ (TaKaRa Bio, Inc., Otsu, Japan). The conditions of denaturation, annealing and extension were as follows: 95 ^°^C for 30 sec, 45 cycles at 95 ^°^C for 5 sec and 60 ^°^C for 20 sec. Relative gene expression was analysed by the 2^- ct^ method and standardized by the GAPDH level. The primers used in the experiment were as follows: GAPDH, forward 5’-GCACCGTCAAGGCTGAGAAC-3’ and reverse 5’-TGGTGAAGACGCCAGTGGAA-3’; ALP, forward 5’-GTGAACCGCAACTGGTACTC-3’ and reverse 5’-GAGCTGCGTAGCGATGTCC-3’; COL1, forward 5’-GCTGATGATGCCAATGTGGTT-3’ and reverse 5’-CCAGTCAGAGTGGCACATCTTG-3’; RUNX2, forward 5’-GTTTCACCTTGACCATAACCGT-3’ and reverse 5’-GGGACACCTACTCTCATACTGG-3’.

***Statistical analysis***

All experiments were repeated at least three times, and the results are expressed as the mean±standard deviation (SD). Prism (version 6.0) software was used for statistical analysis. For the comparison of groups, one-way ANOVA was used to analyse the difference between experimental groups. Statistical significance was accepted when the *P-value* were<0.05.

## Results

***Characterization of the hPDLSCs***

**Figure 1:**
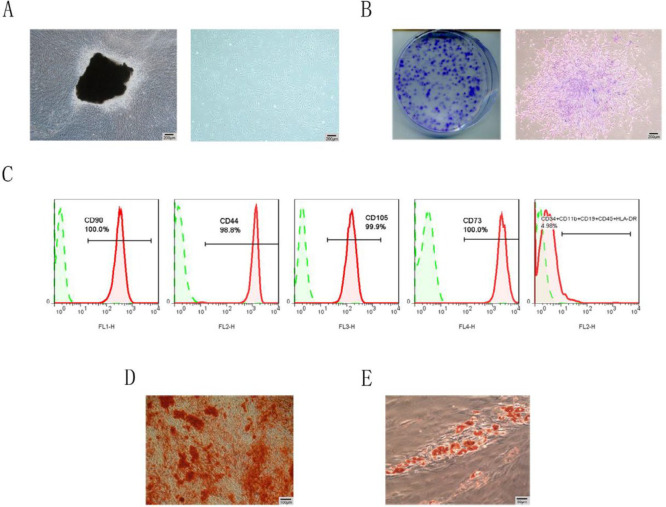
Characterization of hPDLSCs

The hPDLSCs presented a typical spindle-shaped morphology (Figure 1A), and the cells showed a good clonogenic ability (Figure 1B). In flow-cytometry, the hPDLSCs were negative for CD34, CD11b, CD19, CD45 and HLA-DR but were positive for CD73, CD44, CD105 and CD73 (Figure 1C). In addition, Alizarin Red staining showed the formation of mineralized nodules, and Oil Red O staining showed lipid droplet formation (Figure 1D-E). These results indicated that the cells isolated in the study exhibited phenotypic characteristics similar to MSCs.

***Effect of curcumin on cell viability***

**Figure 2:**
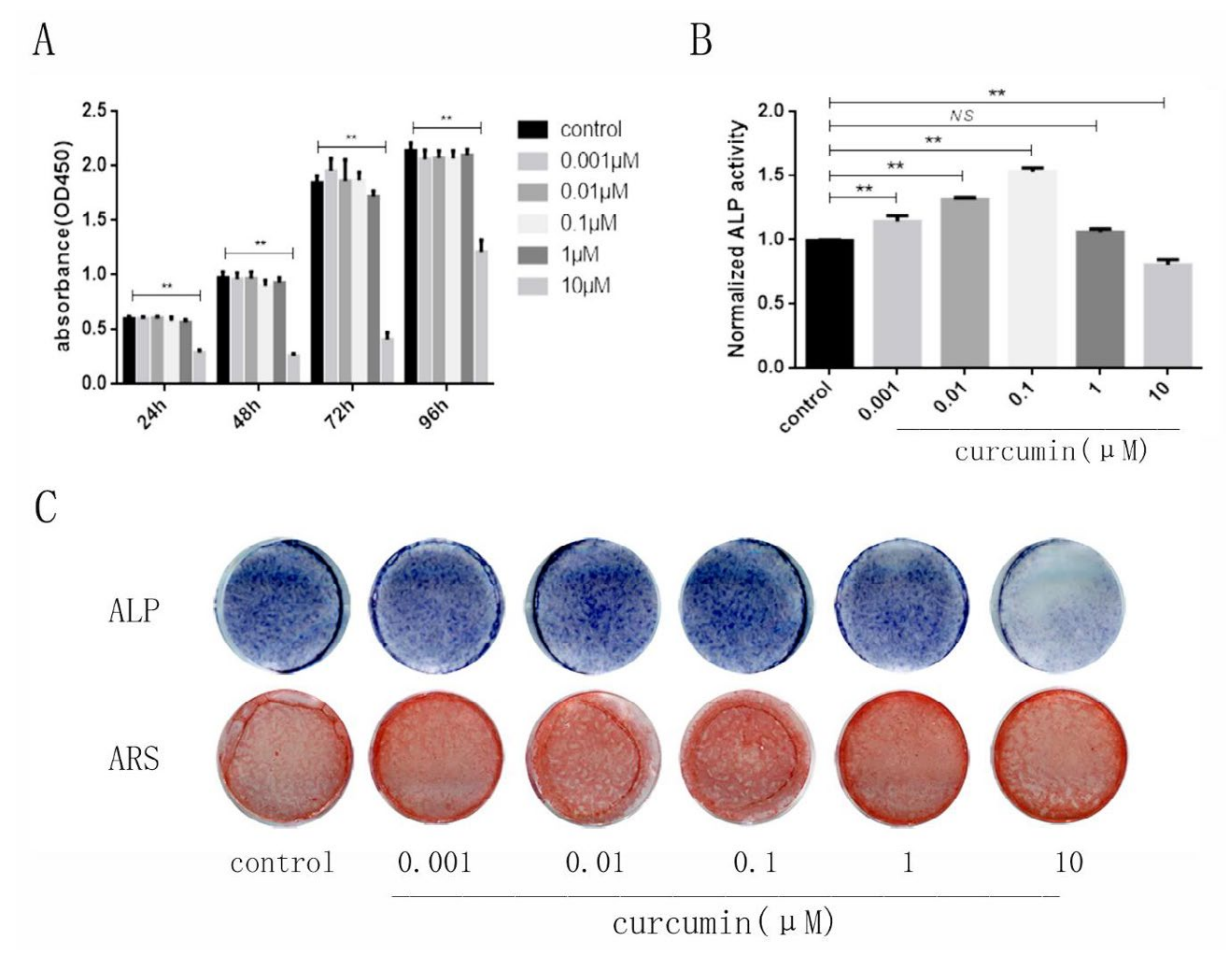
Effects of curcumin on hPDLSCs viability and osteogenic differentiation

To evaluate the toxicity of curcumin on hPDLSCs, we performed a cell viability assay. The results showed that curcumin at low concentrations (0.001 μM, 0.01 μM, 0.1 μM, 1 μM) was nontoxic to cells, and only a high dose of curcumin (>10 μM) inhibited cell viability (Figure 2A).

***Effect of curcumin on osteogenic differentiation***

To further investigate the effect of curcumin on the osteogenic differentiation of hPDLSCs, we induced osteoblast differentiation with different concentrations of curcumin. The results showed that curcumin at low concentrations could significantly increase the ALP activity and calcium content compared to those in the blank control group, and 0.1 μM curcumin had the strongest effects (Figure 2B-C). These results suggested that curcumin at appropriate concentrations could promote the osteogenic differentiation of hPDLSCs.

***Curcumin activates the PI3K/AKT/Nrf2 signaling pathway***

**Figure 3:**
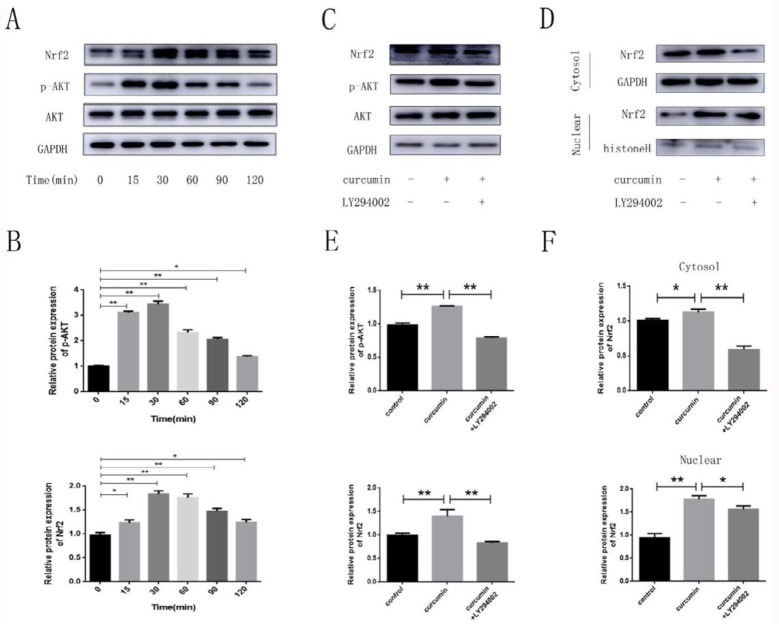
Effects of curcumin treatment on AKT and Nrf2

To clarify whether the promoting effect of curcumin was related to the PI3K/AKT/Nrf2 pathway, we treated hPDLSCs with 0.1 μM of curcumin at different time intervals and then detected the p-AKT and Nrf2 protein levels. All of them were significantly upregulated, especially at 30 min (Figure 3A-B). Next, we pretreated the cells with a PI3K/AKT pathway inhibitor (LY294002). LY294002 significantly reduced the curcumin-induced AKT phosphorylation, whereas the expression levels of total AKT were not changed, indicating that LY294002 efficiently blocked the curcumin-induced activation of the PI3K/AKT pathway. Besides, curcumin exposure dramatically stimulated the translocation of Nrf2 into the nucleus, and these signals were blocked by LY294002 (Figure 3C-F). 

***The PI3K/AKT/Nrf2 signaling pathway is involved in the osteogenic effect of curcumin on hPDLSCs***

**Figure 4.**
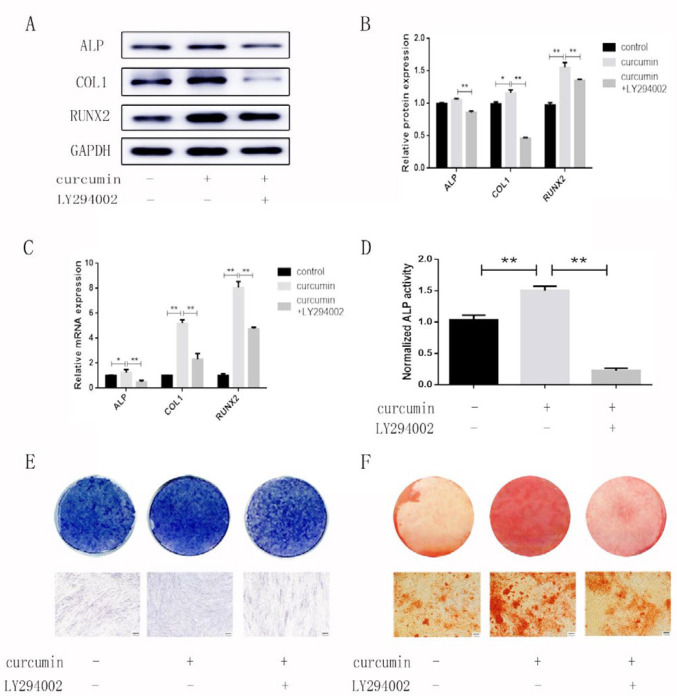
Effects of the PI3K/AKT pathway on the curcumin-mediated osteogenic differentiation of hPDLSCs

**Figure 5:**
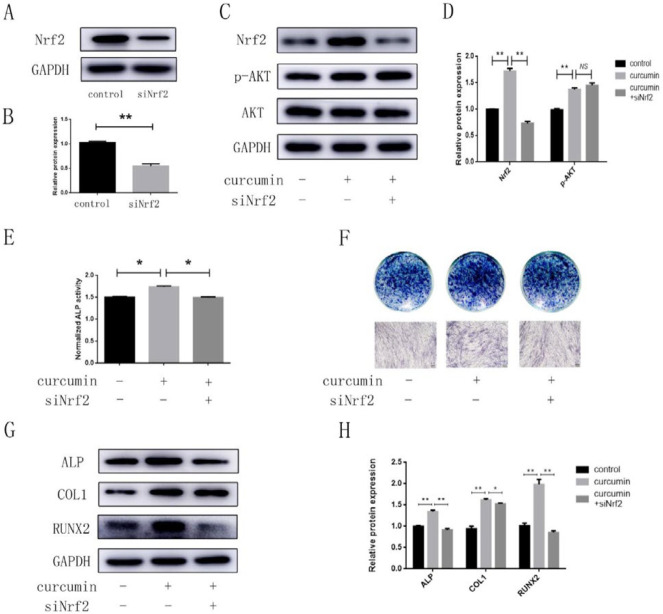
siNrf2 downregulated the effects of curcumin on the osteogenic differentiation of hPDLSCs

To further explore whether the PI3K/AKT/Nrf2 signaling pathway was related to the promoting effect of curcumin on the osteogenic differentiation of hPDLSCs, we established three groups: the control group, the curcumin group and the LY294002+curcumin group. In the curcumin group, the protein and mRNA levels of ALP, COL1, and RUNX2 were obviously higher than those in the other groups (Figure 4A-C). In addition, the results of ALP activity analysis, ALP staining and Alizarin Red staining showed that curcumin exposure dramatically increased the ALP activity and calcium content in the differentiated hPDLSCs (Figure 4D-F). However, the expression of osteogenesis-related indicators in the LY294002+curcumin group showed a downward trend. These results suggested that the curcumin-induced osteogenesis was inhibited when the PI3K/AKT signaling pathway was blocked. To confirm the role of Nrf2 in curcumin-induced bone formation, we constructed a cell model with small interfering RNA and successfully interfered with the Nrf2 gene (Figure 5A-B), and then, we established three groups: the control group, the curcumin group, and the curcumin+siNrf2 group. Western blotting analysis confirmed that a reduction was shown in the total Nrf2 protein level after transfection, while the curcumin-mediated p-AKT induction was not attenuated by transfection with Nrf2 siRNA (Figure 5C-D). In addition, the results of ALP activity analysis, ALP staining and western blotting all showed that the effects of curcumin on osteogenesis were inhibited when the Nrf2 gene was knocked down (Figure 5E-H).

(A) Morphological characteristics of hPDLSCs. (B) Detection of the clonogenic ability. (C) hPDLSCs negatively expressed CD34, CD11b, CD19, CD45 and HLA-DR but positively expressed CD90, CD44, CD105 and CD73. (D) The cells were cultured in osteogenic medium for 21 days and then stained with Alizarin Red. (E) The cells were cultured in adipogenic medium for 28 days and then stained with Oil Red O. hPDLSCs: Human periodontal ligament stem cells

Effect of curcumin at different doses on (A) cell viability, (B) ALP activity, (C) ALP staining after osteogenic induction for 7 days, and Alizarin Red staining for 21 days. *P<0.05, **P<0.01, NS, not significant

hPDLSCs: Human periodontal ligament stem cells, ALP: Alkaline phosphate

(A) The protein expression of Nrf2, p-AKT and AKT at different time periods of treatment with curcumin. (B) Quantification of the western blotting data from A. (C) The protein expression of total Nrf2, p-AKT, AKT and (D) cytosolic and nuclear Nrf2 in the presence or absence of an AKT inhibitor (LY294002). (E) (F) Quantification of the data from C and D. *P<0.05, **P<0.01, NS, not significant

Nrf2: Erythroid transcription factor NF-E2, AKT: Protein kinase B

(A) The protein and (C) mRNA levels of ALP, COL1, and RUNX2 in the presence or absence of LY294002 after osteogenic induction for 7 days. (B) Quantification of the western blotting data from A. (D) ALP activity, (E) ALP staining and (F) Alizarin Red staining in the presence or absence of LY294002 after osteogenic induction medium for 7 days and 21 days. *P<0.05, **P<0.01

hPDLSCs: Human periodontal ligament stem cells, ALP: Alkaline phosphate, PI3K: Phosphatidylinositol-3-kinase, AKT: Protein kinase B

(A) The protein level of Nrf2 when the cells were transfected with nontargeting control siRNA or siNrf2. (B) Quantification of the western blotting data from A. (C) The protein levels of Nrf2 and p-AKT in the presence or absence of siNrf2. (D) Quantification of the western blotting data from B. (E) ALP activity, (F) ALP staining and (G) the protein levels of ALP, COL1 and RUNX2 in the presence or absence of siNrf2 after osteogenic induction for 7 days. (H) Quantification of the western blotting data from G. *P<0.05, **P<0.01, NS, not significant

hPDLSCs: Human periodontal ligament stem cells, ALP: Alkaline phosphate, siNrf2: Erythroid transcription factor NF-E2 siRNA, AKT: Protein kinase B

## Discussion

Curcumin, as a natural phytochemical, has been widely used in research because of its extensive pharmacological activity and trustworthy safety. In recent years, the effect of curcumin on bone formation has received increasing attention. Studies have confirmed that curcumin can promote the osteogenic differentiation of MSCs (15, 26). Bone formation is regulated by multiple signaling pathways, and it is necessary to conduct further investigations to identify whether curcumin could affect the osteogenic differentiation of hPDLSCs through certain pathways. This study was undertaken to explore the effect of curcumin on the osteogenic differentiation of hPDLSCs and its possible mechanism.

In this study, we observed the effects of curcumin at different concentrations on the cell viability and osteogenic differentiation of hPDLSCs. ALP, as a marker of osteogenic differentiation, can reflect the ability of early osteogenic differentiation (27). The occurrence of calcium nodules is the manifestation of osteogenic differentiation and maturation, which reflects the ability of late osteogenic differentiation (28). The results of those osteogenesis-related indicators suggested that curcumin at appropriate concentrations was safe and promoted the osteogenic differentiation of hPDLSCs in a concentration-dependent manner, which was consistent with the previously reported osteogenic effect of curcumin (12, 13, 26).

AKT is an important member of the protein kinase family. It is at the centre of the signaling pathway, which is a biological signal transduction pathway initiated by PI3K, and can be activated by many stresses. In recent years, the PI3K/AKT signaling pathway has been identified as a key regulator of cell proliferation and differentiation (29-31). This axis has also been proven to be involved in the differentiation of osteoblasts on many occasions (32-34). However, it is not clear whether curcumin could promote osteogenesis through the PI3K/AKT axis. Accordingly, the experiment explored whether the PI3K/AKT pathway participated in the curcumin-mediated osteogenic differentiation of hPDLSCs, which provided a theoretical basis for the application of curcumin in osteogenesis. We first treated the hPDLSCs with curcumin at different time points and confirmed that curcumin could activate the PI3K/AKT signaling pathway. LY294002 is an effective inhibitor of PI3K/AKT (35), and it can reduce the expression of phosphorylated AKT (Ser473) (36). Our study proved that LY294002 successfully blocked the PI3K/AKT signaling pathway. Then, we detected the related osteogenic indicators and found that the osteogenesis of hPDLSCs was significantly inhibited with LY294002 pretreatment. These results suggested that curcumin could promote the osteogenic differentiation of hPDLSCs by activating the PI3K/AKT signaling pathway.

In addition, curcumin has been widely approved as an effective activator of Nrf2, which can promote Nrf2 release from the Nrf2-Keap1 complex and transfer to the nucleus (22, 23). Then, Nrf2 binds to the antioxidant responsive element (ARE) sites, leading to the upregulation of antioxidant enzymes (37). In recent years, the role of Nrf2 in stem cell-specific differentiation and maintenance has also been emphasized, and it plays a crucial role in bone homeostasis (24, 38). Further studies showed that the activation of AKT signaling induced Nrf2 activation, while the addition of an AKT inhibitor attenuated Nrf2 nuclear translocation (39, 40). Studies have shown that a mild oxidative stress environment induced by glucose oxidase could promote osteogenic differentiation and mineralization of ESC by activating the Nrf2 signal transduction pathway (41). Evidence has suggested that Nrf2 plays an important role in maintaining MSC stemness and promoting osteogenic differentiation (42).

In our study, both the total protein level and nuclear translocation of Nrf2 were increased by curcumin, while this change was reversed after addition of the PI3K/AKT inhibitor LY294002. In addition, to further prove the effects of Nrf2, we successfully established a siNrf2 cell model. The results showed that curcumin-mediated p-AKT induction was not attenuated by transfection with Nrf2 siRNA. Nrf2, as a downstream signaling molecule of PI3K/AKT, may play a potential role in the curcumin-induced osteogenic differentiation of hPDLSCs, and further studies are necessary to evaluate this hypothesis. Our study showed that the curcumin-induced bone formation was also inhibited when Nrf2 was inhibited. The results suggested that Nrf2 was also involved in the curcumin-induced bone formation. Collectively, PI3K/AKT/Nrf2 was involved in the curcumin-induced osteogenic differentiation.

## Conclusion

The present study confirmed that curcumin treatment enhanced the osteogenic differentiation of hPDLSCs and indicated that this effect, at least to some extent, involves the actions of the PI3K/AKT/Nrf2 signaling pathway. These insights may be valuable for improving the osteogenic differentiation of hPDLSCs.

## References

[1] Polimeni G, Xiropaidis AV, Wikesjo UM (2006). Biology and principles of periodontal wound healing/regeneration. Periodontol.

[2] Maeda H, Tomokiyo A, Fujii S, Wada N, Akamine A (2011). Promise of periodontal ligament stem cells in regeneration of periodontium. Stem Cell Res Ther..

[3] Amani H, Arzaghi H, Bayandori M, Dezfuli AS, Pazoki-Toroudi H, Shafiee A (2019). Controlling Cell Behavior through the Design of Biomaterial Surfaces: A Focus on Surface Modification Techniques. Advanced Materials Interfaces..

[4] Butler MS, Robertson AA, Cooper MA (2014). Natural product and natural product derived drugs in clinical trials. Nat Prod Rep..

[5] Moghadamtousi SZ, Kadir HA, Hassandarvish P, Tajik H, Abubakar S, Zandi K (2014). A review on antibacterial, antiviral, and antifungal activity of curcumin. Biomed Res Int..

[6] Ammon HP, Wahl MA (1991). Pharmacology of Curcuma longa. Planta Med..

[7] Qureshi S, Shah AH, Ageel AM (1992). Toxicity studies on Alpiniagalanga and Curcuma longa. Planta Med..

[8] Lao CD, Ruffin MTt, Normolle D, Heath DD, Murray SI, Bailey JM (2006). Dose escalation of a curcuminoid formulation. BMC Complement Altern Med..

[9] Kim SJ, Son TG, Park HR, Park M, Kim MS, Kim HS (2008). Curcumin stimulates proliferation of embryonic neural progenitor cells and neurogenesis in the adult hippocampus. J Biol Chem..

[10] Kang SK, Cha SH, Jeon HG (2006). Curcumin-induced histone hypoacetylation enhances caspase-3-dependent glioma cell death and neurogenesis of neural progenitor cells. Stem Cells Dev..

[11] Mujoo K, Nikonoff LE, Sharin VG, Bryan NS, Kots AY, Murad F (2012). Curcumin induces differentiation of embryonic stem cells through possible modulation of nitric oxide-cyclic GMP pathway. Protein Cell..

[12] Yang MW, Wang TH, Yan PP, Chu LW, Yu J, Gao ZD (2011). Curcumin improves bone microarchitecture and enhances mineral density in APP/PS1 transgenic mice. Phytomedicine..

[13] Hatefi M, Ahmadi MRH, Rahmani A, Dastjerdi MM, Asadollahi K (2018). Effects of curcumin on bone loss and biochemical markers of bone turnover in patients with spinal cord injury. World Neurosurg..

[14] Bharti AC, Takada Y, Aggarwal BB (2004). Curcumin (diferuloylmethane) inhibits receptor activator of NF-kappa B ligand-induced NF-kappa B activation in osteoclast precursors and suppresses osteoclastogenesis. J Immunol..

[15] Wang N, Wang F, Gao Y, Yin P, Pan C, Liu W (2016). Curcumin protects human adipose-derived mesenchymal stem cells against oxidative stress-induced inhibition of osteogenesis. J Pharmacol Sci..

[16] Luo J, Manning BD, Cantley LC (2003). Targeting the PI3K-Akt pathway in human cancer: rationale and promise. Cancer Cell..

[17] Manning BD, Cantley LC (2007). AKT/PKB signaling: navigating downstream. Cell..

[18] Guntur AR, Rosen CJ (2011). The skeleton: a multi-functional complex organ: new insights into osteoblasts and their role in bone formation: the central role of PI3Kinase. J Endocrinol..

[19] Ulici V, Hoenselaar KD, Agoston H, McErlain DD, Umoh J, Chakrabarti S (2009). The role of Akt1 in terminal stages of endochondral bone formation: angiogenesis and ossification. Bone..

[20] Peng XD, Xu PZ, Chen ML, Hahn-Windgassen A, Skeen J, Jacobs J (2003). Dwarfism, impaired skin development, skeletal muscle atrophy, delayed bone development, and impeded adipogenesis in mice lacking Akt1 and Akt2. Genes Dev..

[21] Tsai KS, Kao SY, Wang CY, Wang YJ, Wang JP, Hung SC (2010). Type I collagen promotes proliferation and osteogenesis of human mesenchymal stem cells via activation of ERK and Akt pathways. J Biomed Mater Res A..

[22] Zingg JM, Hasan ST, Meydani M (2013). Molecular mechanisms of hypolipidemic effects of curcumin. Biofactors..

[23] Yang C, Zhang X, Fan H, Liu Y (2009). Curcumin upregulates transcription factor Nrf2, HO-1 expression and protects rat brains against focal ischemia. Brain Res..

[24] Balogun E, Hoque M, Gong P, Killeen E, Green CJ, Foresti R (2003). Curcumin activates the haem oxygenase-1 gene via regulation of Nrf2 and the anti-oxidant-responsive element. Biochem J..

[25] Wang L, Chen Y, Sternberg P, Cai J (2008). Essential roles of the PI3 kinase/Akt pathway in regulating Nrf2-dependent antioxidant functions in the RPE. Invest Ophthalmol Vis Sci..

[26] Gu Q, Cai Y, Huang C, Shi Q, Yang H (2012). Curcumin increases rat mesenchymal stem cell osteoblast differentiation but inhibits adipocyte differentiation. Pharmacogn Mag..

[27] Harris MT, Butler DL, Boivin GP, Florer JB, Schantz EJ, Wenstrup RJ (2004). Mesenchymal stem cells used for rabbit tendon repair can form ectopic bone and express alkaline phosphatase activity in constructs. J Orthop Res..

[28] Nakai K, Kawato T, Morita T, Yamazaki Y, Tanaka H, Tonogi M (2015). Angiotensin II suppresses osteoblastic differentiation and mineralized nodule formation via AT1 receptor in ROS17/28 cells. Arch Med Sci..

[29] Peltier J, O’Neill A, Schaffer DV (2007). PI3K/Akt and CREB regulate adult neural hippocampal progenitor proliferation and differentiation. Dev Neurobiol..

[30] Meng Q, Xia C, Fang J, Rojanasakul Y, Jiang BH (2006). Role of PI3K and AKT specific isoforms in ovarian cancer cell migration, invasion and proliferation through the p70S6K1 pathway. Cell Signal..

[31] Qiao J, Paul P, Lee S, Qiao L, Josifi E, Tiao JR (2012). PI3K/AKT and ERK regulate retinoic acid-induced neuroblastoma cellular differentiation. Biochem Biophys Res Commun..

[32] Meng YB, Li X, Li ZY, Zhao J, Yuan XB, Ren Y (2015). microRNA-21 promotes osteogenic differentiation of mesenchymal stem cells by the PI3K/beta-catenin pathway. J Orthop Res..

[33] Lu SY, Wang CY, Jin Y, Meng Q, Liu Q, Liu ZH (2017). The osteogenesis-promoting effects of alpha-lipoic acid against glucocorticoid-induced osteoporosis through the NOX4, NF-kappaB, JNK and PI3K/AKT pathways. Sci Rep..

[34] Zhang J, Liu X, Li H, Chen C, Hu B, Niu X (2016). Exosomes/tricalcium phosphate combination scaffolds can enhance bone regeneration by activating the PI3K/Akt signaling pathway. Stem Cell Res Ther..

[35] Walker EH, Pacold ME, Perisic O, Stephens L, Hawkins PT, Wymann MP (2000). Structural determinants of phosphoinositide 3-kinase inhibition by wortmannin, LY294002, quercetin, myricetin, and staurosporine. Mol Cell..

[36] Freudlsperger C, Horn D, Weissfuss S, Weichert W, Weber KJ, Saure D (2015). Phosphorylation of AKT(Ser473) serves as an independent prognostic marker for radiosensitivity in advanced head and neck squamous cell carcinoma. Int J Cancer..

[37] Lee JM, Johnson JA (2004). An important role of Nrf2-ARE pathway in the cellular defense mechanism. J Biochem Mol Biol..

[38] Park CK, Lee Y, Kim KH, Lee ZH, Joo M, Kim HH (2014). Nrf2 is a novel regulator of bone acquisition. Bone..

[39] Wu J, Li Q, Wang X, Yu S, Li L, Wu X (2013). Neuroprotection by curcumin in ischemic brain injury involves the Akt/Nrf2 pathway. PLoS One..

[40] Qi Z, Ci X, Huang J, Liu Q, Yu Q, Zhou J (2017). Asiatic acid enhances Nrf2 signaling to protect HepG2 cells from oxidative damage through Akt and ERK activation. Biomed Pharmacother..

[41] Sim HJ, Kim JH, Kook SH, Lee SY, Lee JC (2016). Glucose oxidase facilitates osteogenic differentiation and mineralization of embryonic stem cells through the activation of Nrf2 and ERK signal transduction pathways. Mol Cell Biochem..

[42] Yoon DS, Choi Y, Lee JW (2016). Cellular localization of NRF2 determines the self-renewal and osteogenic differentiation potential of human MSCs via the P53-SIRT1 axis. Cell Death Dis..

